# Involvement of endoplasmic reticulum stress in rifampicin-induced liver injury

**DOI:** 10.3389/fphar.2022.1022809

**Published:** 2022-10-20

**Authors:** Wanqing Hou, Bernard Nsengimana, Chuyun Yan, Bjorn Nashan, Shuxin Han

**Affiliations:** ^1^ Department of Hepatobiliary Surgery, Anhui Province Key Laboratory of Hepatopancreatobiliary Surgery, The First Affiliated Hospital of USTC, Division of Life Sciences and Medicine, University of Science and Technology of China, Hefei, China; ^2^ Department of Organ Transplantation Center, The First Affiliated Hospital of University of Science and Technology of China, Hefei, China

**Keywords:** rifampicin, hepatocyte toxicity, liver injury, ER stress, adaptive mechanism

## Abstract

Rifampicin is a first-line antituberculosis drug. Hepatocyte toxicity caused by rifampicin is a significant clinical problem. However, the specific mechanism by which rifampicin causes liver injury is still poorly understood. Endoplasmic reticulum (ER) stress can have both protective and proapoptotic effects on an organism, depending on the environmental state of the organism. While causing cholestasis and oxidative stress in the liver, rifampicin also activates ER stress in different ways, including bile acid accumulation and cytochrome p450 (CYP) enzyme-induced toxic drug metabolites *via* pregnane X receptor (PXR). The short-term stress response helps the organism resist toxicity, but when persisting, the response aggravates liver damage. Therefore, ER stress may be closely related to the “adaptive” mechanism and the apoptotic toxicity of rifampicin. This article reviews the functional characteristics of ER stress and its potentially pathogenic role in liver injury caused by rifampicin.

## Introduction

The liver is an essential organ for drug metabolism and elimination; thus, it is also susceptible to drug toxicity. Drug-induced liver injury (DILI) is one of the most challenging liver diseases facing hepatologists and involves damage to hepatocytes and other cells in the liver (Danan and Teschke, 2015). Antibiotics are the top group of drugs that induce liver toxicity, and the clinical manifestations of this toxicity range from nonspecific transaminase elevation to fulminant liver failure (Makhlouf et al., 2008; Bjornsson, 2016). A recent epidemiological analysis in China found that the majority of DILI clinically manifested as hepatocellular injury (51.39%), cholestatic injury (20.31%), and mixed injury (28.30%), and the most common drug type causing drug-induced injury was antituberculosis drugs (21.99%) (Shen et al., 2019). Globally, DILI has become the leading cause of acute *in vivo* liver failure (David and Hamilton, 2010; Iruzubieta et al., 2015; Andrade et al., 2019). In 2019, there were an estimated 10 million new tuberculosis (TB) cases worldwide, of which about 833,000 were in China, accounting for more than 8% and ranking third in the world. About 1.41 million people died due to TB in 2019, of which about 33,000 died in China (Chen et al., 2021). Rifampicin, a widely used first-line drug for TB, has been shown to cause severe liver injury, including cholestasis and hyperbilirubinemia (Capelle et al., 1972; Yew and Leung, 2006; Tostmann et al., 2008; Wondwossen et al., 2016; Su et al., 2021). Rifampicin is a bactericidal drug that inhibits the DNA-dependent RNA polymerase of *mycobacterium tuberculosis* (MTB) by binding to the *β* subunit of the enzyme to inhibit RNA synthesis (Hartmann et al., 1967). The metabolite of rifampicin, desacetylrifampicin, also has antibacterial activity (Donald et al., 2011).

Although rifampicin is mostly metabolized in the liver, the mechanism of hepatotoxicity is not well understood. Emerging evidence suggests that Endoplasmic reticulum (ER) stress activates the unfolded protein response (UPR) and plays a vital role in the pathogenesis of the DILI (Malhi and Kaufman, 2011). Previous studies have shown that rifampicin can cause damage to hepatocyte organelles, including nuclear deformation, chromosome condensation, and rough ER swelling, which can increase the level of nitric oxide and the expression of cytokines like interleukin-8 (IL-8) (Yuhas et al., 2011; Vahdati-Mashhadian et al., 2013). Rifampicin can produce free radicals, which may be related to changes in cytochrome p450 (CYP) enzymes such as CYP2E1, CYP1A1, and CYP1A2 (Lian et al., 2013). Upon entry into cells of mammalian organs such as the liver, kidney, and lung, rifampicin accumulates and causes apoptosis, necrosis, and fibrosis. Meanwhile, recent studies have shown that rifampicin-induced hepatotoxicity is closely related to ER stress, which may affect changes from the stress pathway to the stably regulated ER UPR, a process that protects cells and the homeostasis of which is critical for the cell survival (Guicciardi et al., 2013; Bozaykut et al., 2016). The ER is the primary site for the folding and maturation of transmembrane, secretory, and ER-resident proteins. ER stress can cause the aggregation of unfolded proteins and affect cell survival. To relieve this stress, cells activate the UPR as an intracellular signaling pathway that integrates the folded state of proteins. The UPR includes the regulation of UPR gene transcription, the inhibition of total protein synthesis, and the activation of ER-related protein degradation. All of these processes provide adaptive responses for cell survival. The UPR induces the transcription of genes that mediate the degradation of ER proteins. The components of the UPR stimulate the degradation and clearance of unfolded proteins in the ER lumen. Several target genes encode proteins that stimulate the secretion pathway to reduce the concentration of unfolded proteins (Liu and Kaufman, 2003). The disruption of ER homeostasis leads to the accumulation of unfolded proteins, which threatens all cells (Oakes and Papa, 2015). If protein folding is not restored, the suffering cells will proceed to apoptosis.

It has been found that rifampicin can activate the protein kinase R-like endoplasmic reticulum kinase (PERK)–transcription factor 4(ATF4)– CCAAT/enhancer binding protein homolog protein (CHOP) pathway, which is closely related to the cell damage induced by rifampicin, and CHOP is one of the targets of rifampicin-induced apoptosis (Wang et al., 2021). Moreover, the ER stress inhibitor 4-PBA can inhibit the protein expression and gene expression of the PERK–ATF4–CHOP pathway induced by rifampicin, inhibit cell apoptosis, increase cell survival, and reduce the levels of cell damage biomarkers, which consequently has a cytoprotective effect (Zhang et al., 2016). Therefore, this article reviews the correlation between ER stress and rifampicin hepatotoxicity, which may provide appreciable insights into the future clinical use of rifampicin.

## Rifampicin: Endoplasmic reticulum stress inducer

Rifampicin was introduced in the 1960s as part of a combination chemotherapy regimen for TB. This revolutionized TB treatment by shortening the duration of antituberculosis therapy and increasing cure rates (Maggi et al., 1966; Murray et al., 2015). The antimicrobial effect of rifampicin against MTB and the development of rifampicin resistance are concentration-dependent (Gumbo et al., 2007; Alsultan and Peloquin, 2014). Rifampicin exhibits antimycobacterial effects by interacting with the *β* subunit of RNA polymerase to prevent DNA-directed RNA synthesis in MTB (McClure and Cech, 1978; Rastogi and David, 1993). However, the long-term use of rifampicin can cause abnormal liver function, including significant increases in bile acid and bilirubin levels in the body (Thakkar et al., 2017).

Studies on liver injury induced by rifampicin have been mainly focused on cholestasis, oxidative stress, and mitochondrial damage. Guo et al. reported that the serum biochemical indexes alanine transaminase (ALT), aspartate transferase (AST), alkaline phosphatase (ALP), direct bilirubin, total bilirubin, and total bile acids were significantly increased in rats after continuous gavage with rifampicin for 14 days (Guo et al., 2015). Rifampicin administration significantly reduced the expression of sodium-taurocholate cotransport polypeptide (NTCP) and bile salt export pump (BSEP) in the liver. Therefore, the current studies suggest that the decreased expression of bile acid transporters may mediate the cholestatic liver injury caused by rifampicin. The binding ability of NTCP to isoniazid is closely related to the dose of rifampicin (Ito et al., 2021). Isoniazid is another first-line antituberculosis drug, that is, also hepatotoxic. Isoniazid can be hydrolyzed to form toxic hydrazine, and the process can be induced by rifampicin. When isoniazid and rifampicin are combined for treatment, rifampicin can stimulate hydrazine synthesis, thus increasing the toxic effects of the isoniazid (Ellard et al., 1978). For example, the combination of rifampicin and isoniazid leads to cholestatic liver injury, which is related to the heme metabolism (Brewer et al., 2019). Co-therapy of rifampicin and isoniazid exhibits high levels of CYP enzymes, such as CYP2E1 and CYP2B10 (Brewer et al., 2020). The CYP enzymes can metabolize the isoniazid to produce the toxic metabolite, hydrazine. A high level of hydrazine promotes ROS production, and ROS can then increase the formation of ferric (Fe^3+^) and hydroxyl radicals from ferrous (Fe^2+^) through the Fenton reaction, which promotes heme degradation and leads to liver injury (Tafazoli et al., 2008). Heme degradation increases the protoporphyrin IX level, which subsequently blocks the bile flow. Rifampicin may also induce free-radical production by altering CYP enzymes, and rifampicin alone increases the expression of CYP1A2 (Sun et al., 2019). In addition, the cytochrome P450 enzymes such as CYP3A induced by rifampicin have been implicated in many clinically relevant drug-drug interactions, as this family of enzymes catalyzes metabolic reactions that are the main elimination pathways for most drugs (Yamashita et al., 2013). Inhibition of CYP enzymes can lead to an increase in the blood levels of administered drugs and life-threatening adverse drug reactions (Josephson, 2010). Rifampicin can also mediate apoptosis, necrosis, and fibrosis in mammalian epithelial cells of the organs including the liver, kidney, and lung. Mitochondria may be involved in the apoptosis induced by rifampicin, and mitochondrial disorders produce a large number of free radicals by releasing cytochrome c.

Previous studies have shown that rifampicin can cause cholestasis. The mechanism may be related to changes in the integrity of bile acid transport channels and tight junctions in the hepatocytes (Chen et al., 2009). Many liver diseases, including cholestasis, are closely related to ER stress (Robin et al., 2018). Liver injury caused by cholestasis leads to the accumulation of toxic bile salts in the liver, which can activate the gene expression of glycoregulatory protein 78 (GRP78) and CHOP and induce the UPR (Dara et al., 2011). At the same time, there is increasing evidence that ER stress plays a vital role in drug-induced liver injury. Different concentrations of rifampicin induce liver injury by increasing the ER stress features. Interestingly, the inhibition of ER stress attenuates the cholestatic features (Burban et al., 2018). Tauroursodeoxycholic acid attenuates the rifampicin-induced injury in HepG2 cells by decreasing the ER stress (Zhang et al., 2017). To further examine the involvement of ER stress in liver injury, knockout of sestrin2, a conserved stress-inducible protein, increases apoptosis and liver fibrosis in cholestatic mice (Han et al., 2022). Interestingly, early activation of ER stress reduced hepatic bile acid content by inhibiting CYP7A1, and this inhibition was independent of established FXR-dependent or cytokine-mediated pathways. At the same time, ER stress increased the hepatic expression of MRP3 and BSEP in basolateral bile salt efflux pumps. However, sustained ER stress resulted in the suppression of BSEP and NTCP and promoted the hepatic inflammatory response associated with the cytokine activation (Henkel et al., 2017). Therefore, it is of great significance to further study the occurrence of rifampicin hepatotoxicity and ER stress. In summary, when cholestasis and oxidative stress occur, the process of protein synthesis in hepatocytes is impeded, a large number of damaged proteins are accumulated in the body, and consequently, ER stress is activated.

## Mechanism of rifampicin-induced endoplasmic reticulum stress

**FIGURE 1:**
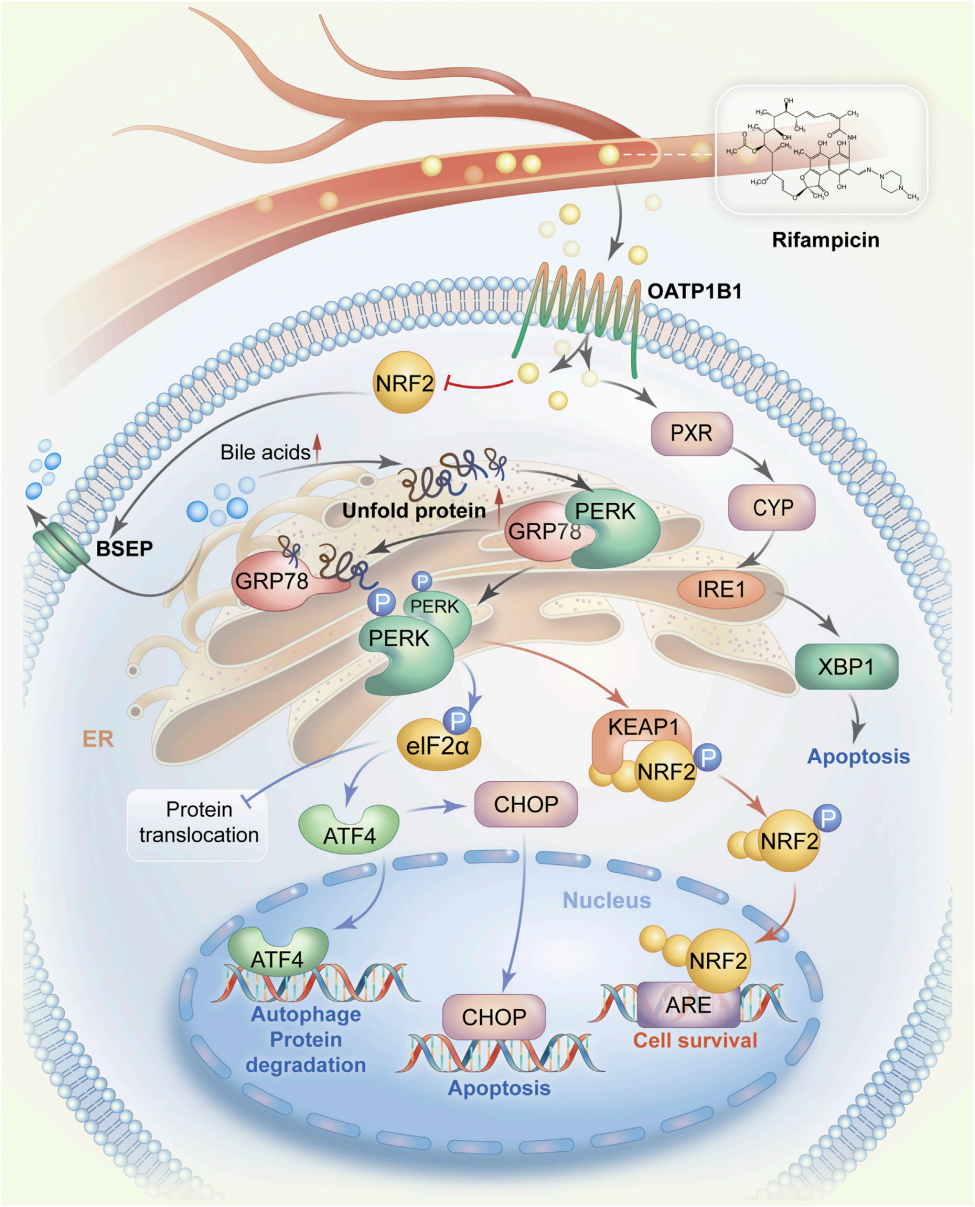
The mechanistic pathways by which rifampicin induces ER stress. Rifampicin is transported to the liver *via* blood circulation and enters hepatocytes *via* OATP1B1. Rifampin can stimulate the expression of CYP enzymes by activating PXR and then promote the IRE1-XBP1 signaling pathway to cause ER stress, resulting in cell apoptosis. On the other hand, after entering hepatocytes rifampicin reduces the expression of BSEP possibly by suppressing the activity of NRF2, leading to the accumulation of bile acids in hepatocytes. Accumulated bile acids interrupt protein folding and cause a large amount of unfolded protein. The unfolded protein competes to bind GRP78 also known as BiP in the GRP78/PERK complex, allowing PERK activation. PERK homodimerization occurs after being phosphorylated. The PERK dimer activates the ATF4-CHOP signaling pathway and promotes cell survival by switching on the NRF2-dependent self-protection mechanism. Abbreviations: ER, endoplasmic reticulum; OATP1B1, organic anion transporting polypeptides1B1; CYP, cytochrome p450; PXR, pregnane X receptor; IRE1, Inositol-requiring enzyme 1; XBP1, X-box binding protein 1; GRP78, glycoregulatory protein 78; PERK, protein kinase R-like endoplasmic reticulum kinase; ATF4, transcription factor 4; CHOP, CCAAT/enhancer binding protein homolog protein; NRF2, nuclear factor E2-related factor 2.

When the body is under the influence of toxic drugs, infection, hypoxia, oxygen stress, and other stressful conditions, the aggregation of misfolded or unfolded proteins in the ER leads to ER stress. The UPR can be activated in the early or middle stages of ER stress and acts as an adaptive protective response to restore the balance of protein folding. However, if the stimulation lasts too long or is too intensive, the UPR will inhibit the adaptive response and induce an apoptosis (Chen and Brandizzi, 2013). The mechanistic pathways of rifampicin-induced ER stress are summarized in Figure 1. Rifampicin can directly induce ER stress by activating the pregnane X receptor (PXR) to stimulate the expression of CYP enzymes. Aberrant expression of CYP enzymes can trigger the ER stress due to the X-box binding protein 1 (XBP1) activation *via* the Inositol-requiring enzyme 1 (IRE1) signaling pathway (Szczesna-Skorupa et al., 2004; Hassani-Nezhad-Gashti et al., 2020). Alternatively, rifampin can also interrupt the bile acid efflux function of BSEP to cause cholestasis and subsequently induce ER stress (Stieger et al., 2000; Byrne et al., 2002). In addition, several previous studies reveal that long-term administration of rifampicin may also inhibit the expression of BSEP, leading to bile acid accumulation in the liver (Chen et al., 2016; Wen et al., 2022). However, the exact underlying mechanism is still not clear. The potent possibility is that rifampicin may suppress nuclear factor E2-related factor 2 (NRF2) transactivation of the BSEP gene expression (Yang et al., 2020; Gao et al., 2021).

Rifampicin can induce the expression of GRP78, which is also known as the immunoglobulin binding protein, and the most abundant ER chaperone molecule (Jing et al., 2014). GRP78 can promote protein folding and assembly, degrade misfolded proteins, and activate ER transmembrane receptors. GRP78 is an essential factor regulating ER homeostasis during ER stress (Hong et al., 2015; Xiong et al., 2015). Studies have shown that hepatic GRP78 plays a crucial role in maintaining ER homeostasis and the viability of hepatocytes, and can inhibit hepatocyte apoptosis. The deficiency of hepatic GRP78 exacerbates liver injury induced by alcohol, high-fat diet, drugs, and toxins (Ji et al., 2011). GRP78 overexpression inhibits ER stress-induced apoptosis, and conversely silencing GRP78 with siRNA activates ER stress-specific caspase cascades (Cao et al., 2012). After activation, GRP78 is dissociated from three membrane receptors in the ER, thereby activating three UPR pathways. Studies have shown that the biological behaviors of the three pathways are very variable in different stages of ER stress. The three UPR pathways are the inositol-demanding kinase 1, PERK, and activating transcription factor-6 pathways, respectively (Ron and Walter, 2007). Under normal conditions, the three ER stress receptor proteins bind to the ER molecular chaperone GRP78 and remain inactivated. When stress occurs, misfolded proteins compete with these receptors to interact with GRP78, dissociating the receptors from GRP78 and activating the unfolded protein response (Zhu and Lee, 2015). When ER stress lasts for a long time, PERK activates eukaryotic translation initiation factor 2α (eIF2α) phosphorylation to promote the nuclear translocation of ATF-4 (Zhang et al., 2019). ATF4 increases the transcription of specific UPR target genes, including CHOP and Tribble’s homolog 3 (TRIB3) genes. Cell apoptosis can be subsequently induced by CHOP (Chen Y. et al., 2014). Eliminating CHOP reduces oxidative stress and protects ER function (Song et al., 2008). The deficiency of CHOP function also reduces the fibrosis of the cholestatic liver. Findings from the mice model show that CHOP knockout decreases the bile acid-induced cell death and attenuates the liver fibrosis caused by cholestatic liver injury (Tamaki et al., 2008). Interestingly, the aforementioned findings reveal that 4-PBA treatment inhibits rifampicin-induced liver injury (L02 cells) by downregulating the ATF4-CHOP pathway. IRE1 has endoribonuclease activity after activation, generating either adaptive or death signals. IRE1 initiates apoptosis or promotes cell survival through the decay of antiapoptotic miRNAs (Avril and Chevet, 2020). IRE1 mediates the splicing process of XBP1 mRNA introns, leading to a frameshift and the introduction of new carboxyl domains during translation, thus becoming a fully functional transcription factor with cytoprotective effects (Coelho and Domingos, 2014). The *in vivo* study has shown that bile acid inducer impairs the expression of hepatic XBP1 and UPR to promote the liver injury (Kriegermeier et al., 2021). The different durations of activation of the above three pathways during ER stress determine cell fate: A short period of activation may be beneficial for cell survival, and, however, when it is continuously activated, the process of protein synthesis in the body is impaired, causing cell death (Lin et al., 2007).

PERK and IRE1α increase mRNA degradation but reduce ER protein folding requirements and inhibit translation, respectively (Song et al., 2017). The regulations in turn reduce the number of unfolded proteins in the ER. IRE1α can trigger different signaling pathways as needed. In the adaptive phase, IRE1α-mediated XBP-1 mRNA splicing activation enhances the transcription of UPR target genes to increase protein folding capacity. IRE1α–XBP1 signaling is attenuated during the transition from adaptation to apoptosis. Meanwhile, IRE1α enhances the intensity of ER stress through the mRNA degradation of particular UPR target genes, including ER chaperones (Grandjean et al., 2020). The expression of PERK was not increased at the initial stage of rifampicin administration but increased at the transcriptional and protein levels after a prolonged administration. Rifampicin induced PERK expression in both dose- and time-dependent manners. These results suggest that there may be several different stages of ER stress after rifampicin administration, such as the adaptation, the transition, and the apoptosis periods.

PERK is a transmembrane protein with its N-terminal region located in the ER lumen, and is sequestrated by GRP78 under unstressed conditions. In response to ER stress, PERK is dissociated from GRP78 and forms a homodimer leading to activation by the self-phosphorylation (Harding et al., 1999). Lin et al. (2009) used chemogenetic strategies to activate PERK dissociation from misfolded proteins and found that persistently activated PERK signaling led to impaired cell proliferation and promoted cell apoptosis. PERK activation promotes the phosphorylation of eIF2α and blocks eIF2α-dependent protein synthesis (adaptation phase) to restore the cellular and molecular homeostasis (Han et al., 2013). The dephosphorylation of eIF2α induces survival signaling and inhibits PERK signaling (Harding et al., 2003). While initially designed to halt global protein translation, eIF2α also selectively increases the translation of some proteins including ATF4. ATF4 increases the production of molecular chaperones promotes ER-associated protein degradation (ERAD), and upregulates many genes like CHOP involved in redox metabolism, amino acid balance, and apoptosis (Liang et al., 2012). CHOP is a transcription factor that can be induced by a variety of non-physiological conditions (Marciniak et al., 2004). Increasing evidence suggests that CHOP-induced apoptosis plays a pivotal role in ER stress (Li et al., 2014). Apoptosis induced by rifampicin is closely related to the activation of the PERK–ATF4–CHOP pathway and particularly CHOP may be one of the main targets.

As a downstream molecule of the PERK pathway in the UPR, NRF2 may play an essential role in regulating oxidative stress and bile acid transporter-mediated adaptive responses. Rifampicin attenuates the survival of liver cancer cells in a dose- and time-dependent manner, and increases biochemical readouts such as LDH, ALT, AST, and ALP in the cell supernatant. At the same time, NRF2 can also increase the expression of bile acid transporter proteins. After knocking down NRF2, the mRNA and protein levels of bile acid transporters were reduced to different degrees and could be restored by the overexpression of the NRF2 (Tanaka et al., 2009). Studies have shown that the PERK–NRF2 pathway is a signaling pathway that promotes cell survival. As a novel substrate of PERK, NRF2 binds to Kelch-like ECH-binding protein 1 (KEAP1) in the cell cytoplasm under normal states. Phosphorylated PERK homodimer causes the dissociation of the NRF2 and KEAP1 complex, and prevents the reformation of the complex as well, and NRF2-absent cells are more likely to die under ER stress (Cullinan et al., 2003). Thus, activating PERK by the UPR is essential for dissociating the NRF2-KEAP1 complex in the cytosol and enables NRF2 to enter into the nucleus functioning as a cell survival transcription regulator.

## Conclusion and perspectives

Rifampicin can mediate liver injury by inducing ER stress in different ways. First, rifampicin is associated with bile acid accumulation. Second, rifampicin can directly induce ER stress by activating PXR-CYP enzymes-XBP1-IRE1 signaling pathway (Szczesna-Skorupa et al., 2004; Hassani-Nezhad-Gashti et al., 2020). Bile acids can promote the increase of intracellular calcium and oxygen reactive stress (Adachi et al., 2014). Both oxidative stress and accumulation of intracellular calcium are known to modulate ER stress. To date, the studies on ER stress and drugs have been mainly focused on the toxicity mechanisms of drugs, that is, the damaging effect of ER stress. However, research on the “adaptive” mechanism of drugs, in which the protective effect of drug-induced ER stress is rather limited. Many drugs have “adaptive” phenomena, including acetaminophen and antituberculosis drugs. Understanding the “adaptive” mechanism can help to avoid unnecessary drug withdrawal and to make beneficial decisions, which has important clinical significance. Therefore, more deeply studying the potent role of ER stress in rifampicin-induced liver injury will help us to understand the pathogenesis of liver damage caused by such drugs, and help us to find effective strategies to prevent or ameliorate DILI. Future studies can further explore the impact of rifampicin metabolites on ER stress in liver injury.

## Abbreviations

ALP, alkaline phosphatase; ALT, alanine transaminase; AST, aspartate transferase; BSEP, bile salt export pump; CHOP, CCAAT/enhancer binding protein homolog protein; CYP, cytochrome; DILI, Drug-induced liver injury; ER, Endoplasmic reticulum; GRP78, glycoregulatory protein 78; IRE1, Inositol-requiring enzyme 1; KEAP1, Kelch-like ECH-binding protein 1; MTB, mycobacterium tuberculosis; NRF2, nuclear factor E2-related factor 2; NTCP, sodium-taurocholate cotransport polypeptide; PERK, protein kinase R-like endoplasmic reticulum kinase; PXR, pregnane X receptor; TB, Tuberculosis; UPR, unfolded protein response; XBP1, X-box binding protein 1.
